# Adult playful individuals have more long- and short-term relationships

**DOI:** 10.1017/ehs.2021.19

**Published:** 2021-03-10

**Authors:** Yago Luksevicius de Moraes, Marco Antonio Correa Varella, Caio Santos Alves da Silva, Jaroslava Varella Valentova

**Affiliations:** Institute of Psychology, University of São Paulo, São Paulo, Brazil

**Keywords:** Short- and long-term relationships, adult playfulness, mate selection, sex differences

## Abstract

Number of romantic/sexual relationships is suggested as a proxy of potential reproductive success. Cross-culturally, both sexes desire playful long-term mates and playfulness predicts relationship quality. It is yet to be tested, however, if playfulness is associated with number of long- and short-term relationships. We hypothesised that specific playfulness dimensions would correlate with the number of lifetime short- and long-term relationships. We expected that lighthearted playfulness would be associated with more short-term relationships, while other-directed playfulness would be associated with the number of long-term relationships. In total, 1191 Brazilian adults (mean age = 28.7 years, standard deviation = 10.2) responded to online sociodemographic questions and a playfulness inventory. Other-directed playfulness positively predicted the number of short-term and long-term partners in men and whimsical playfulness predicted the number of short-term relationships in women. This suggests that playfulness is used by both sexes to compete for access to more and better mates, but in slightly different ways. For the first time, we show that playful adults have more partners and that playfulness can be used as a part of mating strategies.

## Graphical abstract

**Figure:**
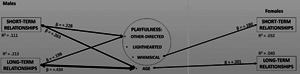

**Social media summary:** Brazilian study shows that playful adults have more partners and playfulness can be used as a part of mating strategies.

## Introduction

Play is ubiquitous in infant and juvenile mammals, having an inverted U-shaped development curve (Bjorklund & Pellegrini, 2002; Lafreniere, 2011), with characteristics commonly found in adaptations selected by Natural Selection (Burghardt, 2005) and by Sexual Selection (Geary, 2003). Despite academic focus in child play, play is a polymorphous phenomenon with traits that are useful in adulthood (Sutton-Smith, 2001). Adults of many species play (Bichara et al., 2018; Burghardt, 2005) and adult play is recognised as a basic human need (Proyer, 2017a; van Leeuwen & Westwood, 2008).

Adult playful behaviour is a manifestation of the psychological concept of playfulness, which is important in some theories regarding personality, motivation and development (Barnett, 2019; Proyer, 2017b). Proyer (2017b, p. 144) defines playfulness as ‘an individual differences variable that allows people to frame or reframe everyday situations in a way such that they experience them as entertaining, and/or intellectually stimulating, and/or personally interesting’. Proyer (2017b) distinguishes four factors of playfulness: (1) other-directed playfulness involves playful social interactions, such as jokes for cheering up or relieving social tension; (2) lighthearted playfulness is about being spontaneous, easygoing and a preference for improvisation over planning; (3) intellectual playfulness is related to a preference for complexity over simplicity, such as riddles and crosswords, and having fun brainstorming and learning difficult issues; and (4) finally, whimsical playfulness is to have fun with odd and unusual things/activities, with being an ‘out-of-the-box person’.

Correlations between playfulness and the Big Five personality traits – i.e. Agreeableness, Conscientiousness, Extraversion, Neuroticism and Openness to Experience – have shown mixed results. According to Barnett (2019), this may have happened because researchers usually do trait-level analyses and disregard individual differences, including gender/sex. She analysed the relationship between playfulness and the 30 facets of the Big Five, which accounted for 52.20% of the male variance in playfulness and 52.70% of the female variance, but only 32.83% of the variance of both sexes mixed (for facet analyses, vs. 21.30% for trait analyses). More playful males have a fast-paced and recurring lifestyle, are gregarious and dutiful, but not very sympathetic, assertive or self-reliant, while playful females are self-reliant, positive, empathetic and open to new experiences, but less imaginative and less assertive than the less-playful ones (Barnett, 2019). Playfulness thus seems to work differently in men and women, indicating a possible role of sexual selection in development and evolution of this personality trait. Indeed, there is a robust cross-cultural evidence on sex differences in infant play that specifically mirror adult Sexual Selection processes of higher male–male competition and higher female parental investment (Byrd-Craven & Geary, 2013).

The Signal Theory of Playfulness suggests that human adult playfulness is a sexual ornament cueing to non-aggressiveness in males and youthfulness (which indicates nubility) in females (Chick, 2001). In the same line, playfulness is a desirable trait in a long-term mate for both men and women (Chick et al., 2012; Proyer & Wagner, 2015).

Playfulness correlates positively with some indicators of physical fitness and health in humans (Proyer et al., 2018b) and it seems to be heritable in some non-human mammals (Siviy, 2016; Svartberg and Forkman, 2002; Walker & Byers, 1991), supporting the hypothesis that playfulness partly evolved through Sexual Selection. Chick et al. (2020) further showed that playfulness is subject to assortative mating, which means that mates are more similar to each other when it comes to playfulness than would be expected if such pairings were random (for review on assortative mating, see Štěrbová & Valentova, 2012).

Playfulness is positively linked to relationship quality, and lighthearted playfulness is the only dimension of the OLIW model that has not shown correlations with relationship satisfaction (Proyer et al., 2019) or with long-term-oriented love styles (Proyer et al., 2018a). Supposedly, lighthearted playfulness is impulsive and present-oriented (Proyer, 2017b). Therefore, lighthearted playfulness may be connected to short-term relationships. Possible link of playfulness to short-term relationships is yet to be tested.

## Short- and long-term relationships

Humans have evolved a repertoire of possible mating strategies, including long- and short-term mating, that vary inter- and intra-individually. Long-term relationships are ‘characterized by high levels of commitment, pair bonding, and emotional involvement, while short-term matings such as one-night stands, brief hookups, and temporary liaisons tend to lack these features (for review see, Buss & Schmitt, 2019). The relationships along the temporary continuum may serve different functions, and both can be adaptive under specific conditions. The overall lifetime number of sexual partners is suggested as a satisfactory metric to infer the potential fitness level of individuals since it demonstrates that they have been accepted or chosen several times as mates (e.g. Faurie et al., 2004). The number of sexual partners throughout life is influenced by many factors, including individual characteristics.

Faurie et al. (2004) and Raymond et al. (1996) suggest that sports represent a form of play that is directly linked to the increase in the biological fitness of individuals. To test the hypothesis, Faurie et al. (2004) conducted a study comparing the number of sexual partners reported by university students, dividing them into groups of athletes and non-athletes. Athlete-students had more sexual partners than non-athletes. Male athletes had up to 94% more sexual partners than non-athletes (2.41 vs. 1.24). Among women, athletes on average had 42% more partners compared with non-athletes (1.6 vs. 1.12). The skill level gave extra help; for example, those who competed in international events had more sexual partners than those who participated only in regional competitions. Thus, the number of sexual partners is influenced by individual physical abilities and achievement. Further, participants with high self-monitoring report a greater number of sexual partners and are more open to experiences without commitment (Snyder et al., 1986). Individuals with high levels of self-monitoring display pronounced behavioural responsiveness to social and interpersonal cues of situational appropriateness. They enjoy being the centre of attention in social situations, using self-dramatisation and exhibitionism to achieve that, are more prone to talk to strangers and can establish new social contacts easily (Snyder et al., 1986).

Similarly, the number of lifetime sexual partners is related positively to extraversion and emotional stability and negatively to agreeableness, with extroversion being the best positive predictor of the number of sexual partners (Rogowska et al., 2020). An extroverted individual seeks companionship from other individuals and according to the authors, this offers a bigger opportunity to find sexual partners.

However, studies rarely address the associations between personality and short-term vs. long-term mating. Short-term mating, including number of short-term partners, is specifically related to high extraversion and the Dark Triad traits (psychopathy, narcissism and machiavellianism), while long-term mating, including number of long-term partners, is associated with high agreeableness, consciousness, openness and low Dark Triad traits (Holtzman & Strube, 2013; Jonason et al., 2009; Valentova et al., 2020).

Furthermore, the strategies applied to attract a short-term mate differ from those used to attract a long-term mate, and are context-sensitive to mate value, operational sex ratio, parasite prevalence, social norms and other factors. For example, both sexes value physical attractiveness more for one-night stands than for marriage, whereas men robustly value youthfulness in women for marriage and women value economic resources in prospective husbands (Buss & Schmitt, 2019).

Playfulness has been suggested as a personality trait functioning as a sexual ornamentation desired in long-term mates (Chick et al., 2012; Proyer & Wagner, 2015). However, to our knowledge, no study has tested if and how playfulness relates to long- and short-term mating strategy of the individual.

## Aims of the current study

This study investigates a possible relationship between playfulness and short- and long-term mating. We hypothesize that, besides its other proximal and distal functions, playfulness may be an adaptation to attract mates. Specifically, we aim to investigate what dimensions of playfulness can predict number of romantic/sexual partners, if it differs depending on the type of relationship (short-term vs. long-term) and if there is any sex/gender difference. We expect that: (h_1_) since playfulness is a desirable trait in long-term mates, all dimensions of playfulness would correlate with the number of romantic/sexual relationships; (h_2_) lighthearted playfulness is about improvisation and lack of planning – we might thus expect it would be positively correlated with number of short-term relationships; and (h_3_) in previous studies, other-directed, intellectual and whimsical playfulness predicted relationship satisfaction, so it is expected that it would positively predict number of long-term relationships.

## Methods

### Participants

The participants were recruited from several social media groups aimed at games as well as through our institution's communication channels, including the university newspaper. In total, 1,191 participants responded online questionnaires; 646 were cisgender men (54.2%, mean age = 27.85 years, standard deviation, SD = 9.40), 494 cisgender women (41.5%, mean age = 29.94, SD = 11.18), 25 transwomen (2.1%, mean age = 27.92, SD = 11.86), nine transmen (0.8%, mean age = 34.67, SD = 11.48), nine females with nonbinary gender identity (0.8%, mean age = 25.22, SD = 3.99) and eight males with nonbinary gender identity (0.7%, mean age = 22.88, SD = 3.60). Because sex/gender was one of the important factors tested in this study and we obtained a relatively small sample of trans and non-binary individuals, we performed the final analyses with the cisgender sample only.

Most participants of the final sample self-reported as white (*n* = 798, 70.0%), exclusively heterosexual (*n* = 713, 62.5%), from the southeastern region of Brazil (*n* = 902, 79.1%) and as having at least started college (*n* = 1,005, 88.2%). The most frequent family income ranged from approximately USD 200.00 to 600.00 monthly (*n* = 284, 24.9%), which corresponds in most part to the lower class, followed by an income ranging from USD 600 to 1,000 monthly (*n* = 254, 22.3%), which corresponds to the lower-middle class. For the full description of the cisgender sample, please refer to the Supplementary Material S1.

### Material (instruments)

The OLIW questionnaire was translated to Portuguese by the first author, then back-translated by another bilingual person, and the other authors compared the back-translation with the original, suggesting improvements.

The OLIW (Proyer, 2017b) consists of 28 statements that measure adult playfulness. It is composed of four facets, each consisting of seven statements: (a) *other-directed playfulness* measures the usage of playfulness in social interactions (e.g. ‘I can use my playfulness to do something nice for other people or to cheer them up’); (b) *lighthearted playfulness* measures improvisation and lack of seriousness (e.g. ‘Many people take their lives too seriously; when things don't work you just have to improvise’); (c) *intellectual playfulness* involves playing with ideas and a preference for complexity (e.g. ‘If I want to develop a new idea further and think about it, I like to do this in a playful manner’); and (d) *whimsical playfulness* embraces enjoyment for unusual and odd things (e.g. ‘I have the reputation of being somewhat unusual or flamboyant’). Responses are on a seven-point Likert-type scale ranging from 1 ‘strongly disagree’ to 7 ‘strongly agree’. This results in four scores ranging from 7 to 49.

The values of Cronbach's *α* in this study were satisfactory for *other-directed* (0.77), *lighthearted* (0.71) and *whimsical* (0.71) and unsatisfactory for *intellectual* (0.49) dimension of playfulness. The original OLIW values of *α* (Proyer, 2017b) ranged from 0.66 to 0.79 in all dimensions in all three samples, with the exception of the three-item version of the other-directed playfulness used in the third sample (*α* = 0.56). Therefore, our intellectual playfulness *α* was inferior to the original ones, while the other dimensions had similar values of *α* to those in the original study. Since the inclusion/exclusion of intellectual playfulness had no effects in the final regressions, we report the correlation between intellectual playfulness and the other variables in Table 1, for the sake of transparency, but omit it from final regression analyses.

**Table 1.** Kendall non-parametric correlations between playfulness and number of romantic/sexual relationships

		1	2	3	4	5	6
1. Other-directed playfulness	Tau		0.246***	0.250***	0.074*	0.160***	0.150***
	*N*		557	557	557	516	516
2. Lighthearted playfulness	Tau	0.262***		0.227***	0.189***	0.098**	0.070*
	*N*	446		557	557	516	516
3. Intellectual playfulness	Tau	0.273***	0.281***		0.123***	0.058	0.110**
	*N*	446	446		557	516	516
4. Whimsical playfulness	Tau	0.210***	0.286***	0.227***		0.039	−0.009
	*N*	446	446	446		516	516
5. Short-term relationships	Tau	0.124***	0.137***	0.098**	0.133***		0.437***
	*N*	416	416	416	416		516
6. Long-term relationships	Tau	0.028	0.064	0.049	0.058	0.309***	
	*N*	416	416	416	416	416	

Note: Results for men are above the diagonal, results for women below the diagonal.

* *P* < 0.05, ** *P* < 0.01, *** *P* < 0.001.

#### Romantic/sexual relationships

We asked the participants how many short-term relationships – defined as casual dates without the expectation of remaining together – and how many long-term relationships – defined as relationships with the expectation of remaining together – the participants had had in their life.

### Procedure

The questionnaires were distributed online via Qualtrics (Provo, UT) platform from November 2019 to July 2020. Most participants were recruited before the COVID-19 pandemics outbreak in March 2020. The participants firstly read and agreed to the informed consent, and then accessed the questionnaires. Participation was voluntary, not financially rewarded and took around 30 min. This specific study was part of a larger project and only questionnaires relevant to this study are presented. The sociodemographic questionnaire was always the first to be answered, and the other ones were randomised. The order of all questions within questionnaires was randomised. Ethical approval was received from the IRB of the Institute of Psychology, University of Sao Paulo, report number 3.597.292.

### Analyses

SPSS version 21 (IBM Corp, Armonk, NY, USA) was used for the analyses. Mostly the data were not normally distributed. We firstly excluded participants who had completed 6% or less (one part of the sociodemographic questionnaire) of the whole survey. We then explored the data with non-parametric Kendall correlations, separately for cis men and cis women. Further, because men were significantly younger than women (*t* = 3.34, d.f. = 956, *P* < 0.001), age entered the subsequent analyses. To test for sex differences, we ran a multivariate general linear model (GLM), with sex/gender as a factor, all other measures as dependent variables and age as a covariate. The effect size estimator employed is Cohen's *d*.

Finally, we ran four linear stepwise regression models, two for men and two for women, with number of short-term relationships and the number of long-term relationships as dependent variables, and the playfulness dimensions and age as predictors. To avoid type I error inflation, and considering we have four regression models, we used probability of *F* entry 0.0125 (= 0.05/4) as the stepping method criteria.

## Results

### Correlations among number of relationships and playfulness

Number of short-term relationships positively correlates with all dimensions of the playfulness dimension (OLIW) in both sexes, except with intellectual and whimsical playfulness for men. Number of long-term relationships correlated positively with other-directed, lighthearted and intellectual playfulness in men but showed no significant correlations with any dimension in women (see Table 1 for correlations, and for descriptive statistics, see Supplementary Material S2).

### Sex differences

The between-subject effects of the multivariate general linear model, controlling for age, revealed significant, although mostly small, sex difference in nearly all variables (see Table 2). On average, men scored higher than women on lighthearted and whimsical playfulness, and women reported having had more long-term relationships than men. There was, however, no sex difference in number of short-term relationships.

**Table 2.** Sex differences in playfulness and sexual strategy

	*F*	*P*	Partial *η*^2^	Men (*N* = 516), mean (SD)	Women (*N* = 416), mean (SD)	Cohen's *d*
Other-directed playfulness	0.339	0.560	0.000	36.1(7.5)	36.2(7.7)	0.01
Lighthearted playfulness	8.087	0.005	0.009	27.9(7.3)	26.5(7.6)	0.19
Whimsical playfulness	8.690	0.003	0.009	29.3(7.1)	27.8(7.8)	0.19
Short-term relationships	0.084	0.772	0.000	6.4(7.0)	6.7(6.6)	0.03
Long-term relationships	3.847	0.050	0.004	1.7(1.7)	2.0(1.6)	0.20

Because of these differences and because it is expected that playfulness works differently in each sex, we further performed regression models separately for men and women.

### Playfulness predictors of numbers of short- and long-term relationships

In men, the regression model was significant for both short-term (*R* = 0.33, *R*^2^ = 0.11, *F* = 31.0, *P* < 0.001) and long-term relationships (*R* = 0.47, *R*^2^ = 0.22, *F* = 73.813, *P* < 0.001). Men's number of short-term relationships was positively predicted by age (*B* = 0.19, SE = 0.03, *t* = 6.06, *P* < 0.001) and other-directed playfulness (*B* = 0.22, SE = 0.04, *t* = 5.57, *P* < 0.001). Likewise, the number of long-term relationships was positively predicted by age (*B* = 0.08, SE = 0.01, *t* = 11.38, *P* < 0.001) and by other-directed playfulness (*B* = 0.05, SE = 0.01, *t* = 5.31, *P* < 0.001).

In women, both regression models were significant but weak (short-term, *R* = 0.20, *R*^2^ = 0.04, *F* = 16.95, *P* < 0.001; long-term, *R* = 0.18, *R*^2^ = 0.04, *F* = 14.45, *P* < 0.001). Women's number of short-term relationships was positively predicted only by whimsical playfulness (*B* = 0.16, SE = 0.04, *t* = 4.12, *P* < 0.001), while the number of long-term relationships was positively predicted only by age (*B* = 0.03, SE = 0.01, *t* = 3.80, *P* < 0.001).

## Discussion

Here we tested if different dimensions of adult playfulness predict variation in the number of short-term and long-term romantic/sexual relationships. The significant correlations between all OLIW's dimensions with number of short- or long-term relationships in men and with short-term relationships in women was positive, corroborating studies suggesting that playful people might be more desirable mates (Chick et al., 2012; Proyer & Wagner, 2015). Playfulness thus can serve directly as a tactic to attract/maintain sexual partners, or indirectly to set the stage for entertainment and flirting. Importantly, different dimensions of playfulness were associated with number of sex partners in men and in women.

Other-directed playfulness was associated with the number of short- and long-term partners in men. This dimension of playfulness is defined as ‘enjoying to play with others; using one's playfulness to make social relations more interesting or to loosen up tense situations with others; enjoying good-heartedly teasing’ (Proyer, 2017b). Thus, as hypothesised by the Signal Theory of Playfulness (Barnett, 2019; Chick, 2001), more other-directed playful males are expected to be less aggressive and more cooperative, and therefore would be more attractive as both a short- and a long-term mate.

On the other hand, whimsical playfulness predicted only short-term relationships in women, but not in men. This dimension correlates positively with fascination towards one's partner and with engagement in maintaining the relationship (Proyer et al., 2018a). Thus, it would be expected that this dimension would predict more long-term relationships, which would be true for women if we kept *P* < 0.05 as the significance threshold.

More investigation is needed about role of whimsical playfulness in mate selection. Perhaps whimsical women are more likely to start a short-term relationship and try to change it into a long-term relationship, but their efforts have the opposite effect in many cases. Theories about the evolutionary benefits for women's engagement in short-term relationships include the opportunity to evaluate potential long-term mates and trade up to a partner of higher mate value (Buss & Schmitt, 2019). Therefore, whimsical women may trade partners until they find one considered a good long-term mate. This is supported by the number of short-term relationships correlating positively with the number of long-term relationships in both sexes. The development of relationships from short to long term should be addressed more by future studies.

The positive correlation between the number of short- and long-term relationships suggests that short- and long-term relationships are not opposites, but rather that individuals who tend to have more long-term relationships also tend to have more short-term relationships. Playfulness was thus a good model to show the validity of previous studies asking only about the number of sexual partners, without specifying short- and long-term context. Multiple mating and concurrent relationships are present cross-culturally, although with more or less stigmatisation (Scelza & Prall, 2018). Thus, short- and long-term mating are not opposite mutually exclusive strategies (Valentova et al., 2020). However, although present in both sexes, the strategies are linked to playfulness differently in men and women, as predicted by the Signal Theory of Playfulness (Chick, 2001; Barnett, 2019).

Furthermore, whimsical playfulness is about being flamboyant and unusual (Proyer, 2017b), which are characteristics typical of the arts (Dissanayake, 2018). Indeed, Varella et al. (2017) show evidence that artisticality may have evolved as a strategy for female intrasexual competition. It fits their Competitive Ornamentation Model (Varella et al., 2017), suggesting that women exhibiting playful tendencies to show off and stand out would compete more for access to more and better mates. Possibly, whimsical playfulness might signal youth and health in women, as suggested by the Signal Theory of Playfulness (Chick, 2001), although this would need to be tested more directly.

Men scored significantly higher on lighthearted playfulness, but it did not predict the number of short- nor of long-term relationships in either sex. According to Proyer et al. (2019), participants who score higher in this dimension are more likely to experience both higher fascination and higher mistrust towards their partners. It seems that some parts of this dimension may contribute to relationships, while others are detrimental (Proyer et al., 2018a).

It is important to note that the models explained 11% of men's variance in number of short-term relationships and 22% of men's variance in number of long-term relationships, but only 4% of women's variances in the number of both short- and long-term relationships. Therefore, men's fitness may be more impacted by playfulness than women's, explaining why, in general, men scored higher than women in OLIW. In this study, sex/gender differences in all playfulness dimensions were small, similarly to those in Proyer (2017b) and smaller than those in Proyer et al. (2018a).

### Limitations of the study

This study relies on self-reports, so it is limited as to how honestly and accurately the participants answered the survey. Another possible limitation would be related to the short- vs. long-term relationship definition, where some participants may have answered based on how long the relationship actually lasted and others answered based on what their expectation was, even if the duration is contrary to the expectation. Future comparisons with other definitions of short- and long-term relationships are recommended.

Also, despite our efforts to include a diverse sample through social media, it is clear we have an over-representation of students from a public university in Brazil's southeast which does not represent Brazilian population heterogeneity. Moreover, more than two-thirds of the data was collected between December and February, highly festive summer months in Brazil, and a few participants answered the questionnaires during the first phase of the COVID-19 pandemic in Brazil, so the extraordinary situations could have influenced the results.

Further, our study is cross-sectional, and we are aware that correlations do not imply a causal relation between the variables. Several correlations may be confounded. Replications using experimental or longitudinal designs are desirable, particularly among different populations. On the other hand, this study is the first of our knowledge to address the relation among subdimensions of playfulness and number of short- and long-term relationships, and provides insights for investigating the proximal and distal causes of playfulness as a part of sexual strategy.

## Conclusions

We have shown that men with high other-directed playfulness have more romantic/sexual relationships throughout their life, even controlling for age, while whimsical playfulness predicted more short-term relationships in women. This suggests that men may use pro-social forms of playfulness which can attract potential mates, as predicted by the Signal Theory of Playfulness, while women may exhibit flamboyant playful tendencies and achieve the same goal, which fits the Competitive Ornamentation Model.

Furthermore, lighthearted playfulness was not a good predictor of number of relationships for either sex, perhaps because some of its traits are beneficial for relationships, such as being good-humoured, while others are probably detrimental, such as carefreeness. This dimension may have different functions or its attractiveness depends on various conditions, such as age, socio-economic status, ethnicity or other personality traits.

There is some evidence that playfulness is desirable in potential long-term mates, and that playfulness improves relationship satisfaction. Our study provided evidence that playful people also have more relationships throughout the lifespan. Future studies might analyse whether and how different dimensions of playfulness affect flirting, acceptance of casual sex, extramarital affairs, mate poaching, and also non-sexual behaviour. For example, playfulness might facilitate making friendships, which could give access to more potential mates through friends. Playfulness may also facilitate interactions with children, being a reliable sign of good parenting. Research on playfulness is a fertile field for social and evolutionary scientists, especially considering how recently we have started to investigate different kinds of playfulness in adults.

## Data Availability

The data that support the findings of this study are openly available at https://osf.io/d9s8u/

## Supplementary material

For supplementary material accompanying this paper visit https://doi.org/10.1017/ehs.2021.19. click here to view supplementary material
