# Supplementary material for: Adult playful individuals have more long- and short-term relationships
Source: Evol Hum Sci. 2021 Mar 10;3:e24. doi: 10.1017/ehs.2021.19 (PMC10427296; doi:10.1017/ehs.2021.19)

Descriptive statistics:

- Other-directed playfulness:


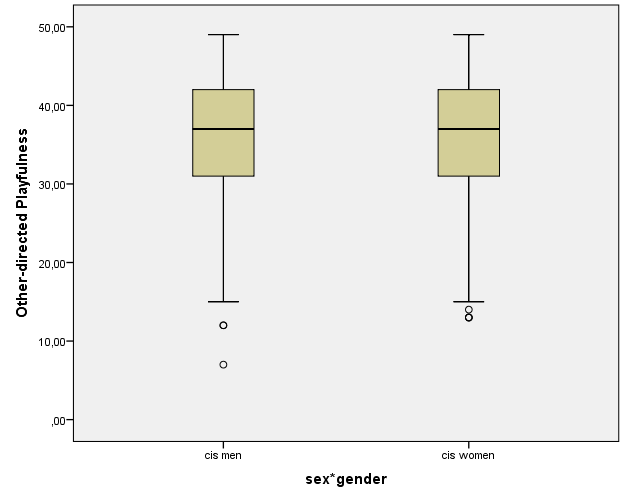

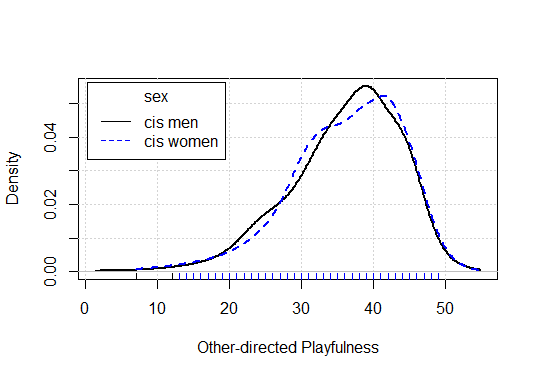


- - Men:
    - *n* = 557
    - Mean = 36.08
    - 95% Lower Bound Confidence Interval for Mean = 35.45
    - 95% Upper Bound Confidence Interval for Mean = 36.71
    - Median = 37.00
    - Standard Deviation = 7.61
    - Minimum = 7.00
    - Maximum = 49.00
    - Interquartile Range = 11.00
    - Skewness = -.661 (Standard Error = .104)
    - Kurtosis = .162 (Standard Error = .207)
  - Women:
    - *n* = 446
    - Mean = 36.27
    - 95% Lower Bound Confidence Interval for Mean = 35.56
    - 95% Upper Bound Confidence Interval for Mean = 36.98
    - Median = 37.00
    - Standard Deviation = 7.61
    - Minimum = 13.00
    - Maximum = 49.00
    - Interquartile Range = 11.00
    - Skewness = -.647 (Standard Error = .116)
    - Kurtosis = .090 (Standard Error = .231)
  - Total:
    - *n* = 1047
    - Mean = 36.09
    - 95% Lower Bound Confidence Interval for Mean = 35.63
    - 95% Upper Bound Confidence Interval for Mean = 36.56
    - Median = 37.00
    - Standard Deviation = 7.62
    - Minimum = 7.00
    - Maximum = 49.00
    - Interquartile Range = 11.00
    - Skewness = -.641 (Standard Error = .076)
    - Kurtosis = .086 (Standard Error = .151)


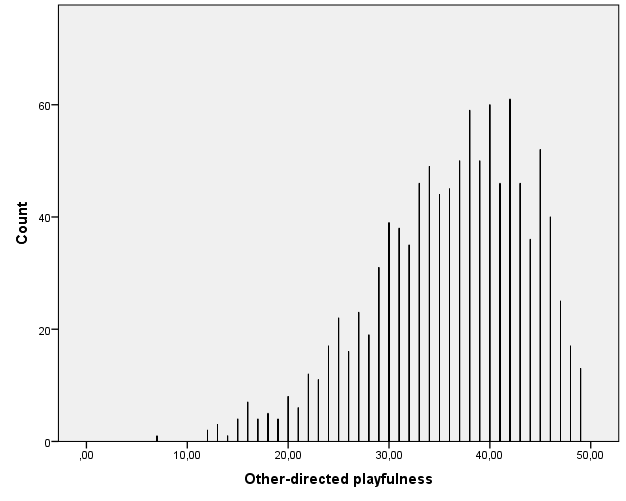


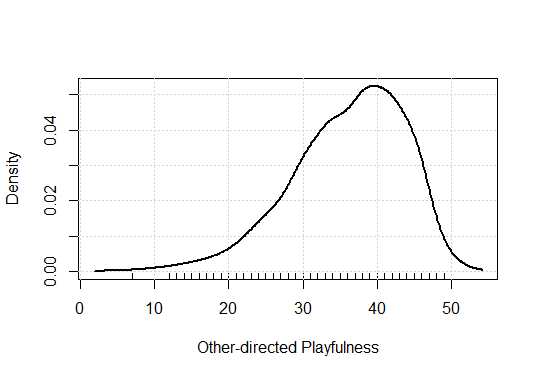


- Lighthearted playfulness:


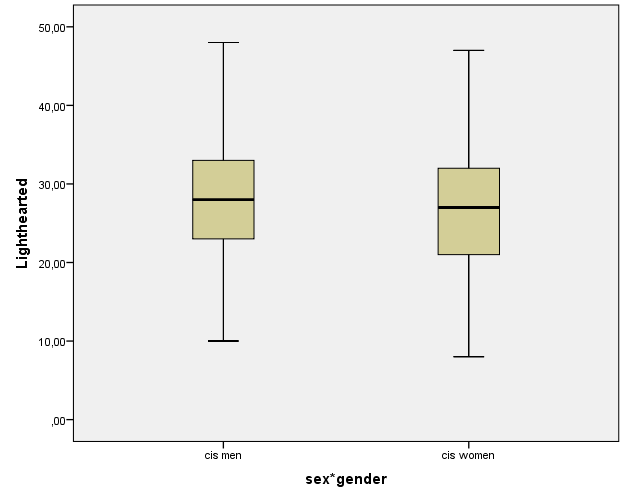


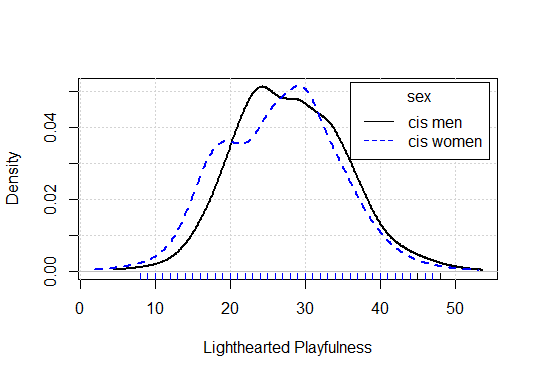


- - Men:
    - *n* = 557
    - Mean = 28.00
    - 95% Lower Bound Confidence Interval for Mean = 27.90
    - 95% Upper Bound Confidence Interval for Mean = 28.61
    - Median = 28.00
    - Standard Deviation = 7.35
    - Minimum = 10.00
    - Maximum = 48.00
    - Interquartile Range = 10.00
    - Skewness = .175 (Standard Error = .104)
    - Kurtosis = -.363 (Standard Error = .207)
  - Women:
    - *n* = 446
    - Mean = 26.59
    - 95% Lower Bound Confidence Interval for Mean = 25.88
    - 95% Upper Bound Confidence Interval for Mean = 27.31
    - Median = 27.00
    - Standard Deviation = 7.70
    - Minimum = 8.00
    - Maximum = 47.00
    - Interquartile Range = 11.25
    - Skewness = .029 (Standard Error = .116)
    - Kurtosis = -.487 (Standard Error = .231)
  - Total:
    - *n* = 1047
    - Mean = 27.44
    - 95% Lower Bound Confidence Interval for Mean = 26.98
    - 95% Upper Bound Confidence Interval for Mean = 27.89
    - Median = 27.00
    - Standard Deviation = 7.52
    - Minimum = 8.00
    - Maximum = 48.00
    - Interquartile Range = 11.00
    - Skewness = .071 (Standard Error = .076)
    - Kurtosis = -.357 (Standard Error = .151)


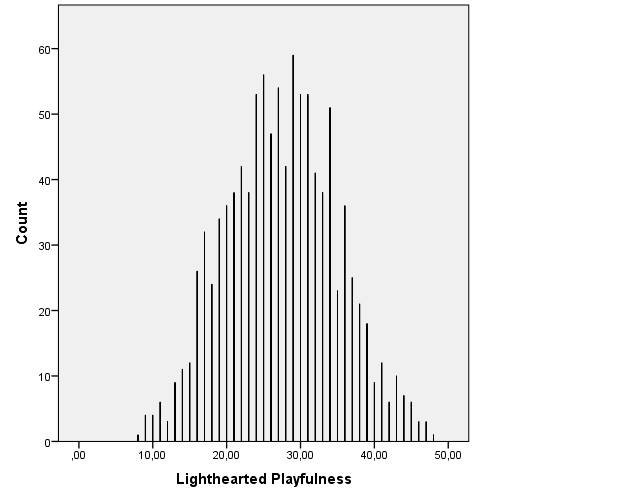


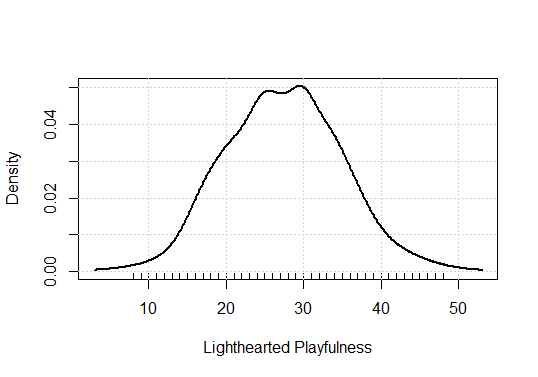


- Intellectual playfulness:


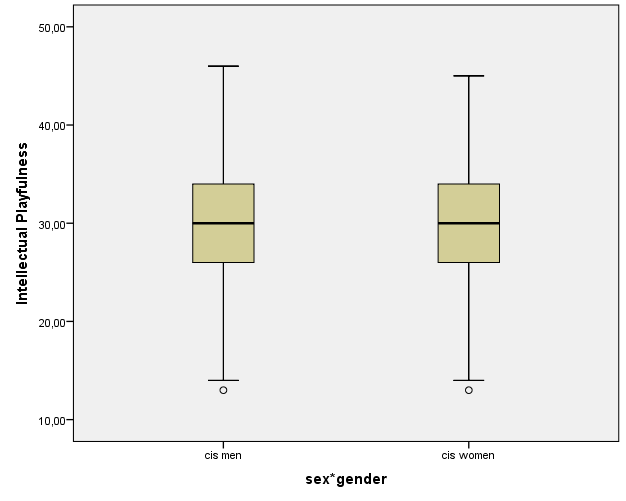


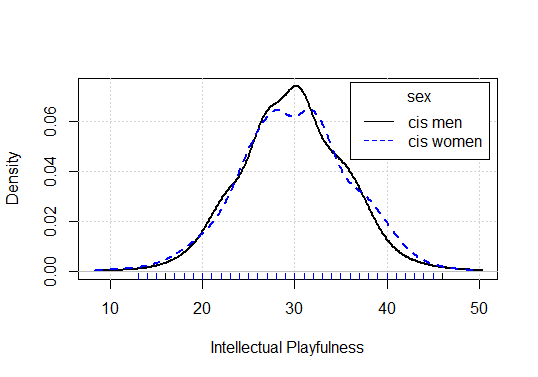


- - Men:
    - *n* = 557
    - Mean = 29.77
    - 95% Lower Bound Confidence Interval for Mean = 29.30
    - 95% Upper Bound Confidence Interval for Mean = 30.24
    - Median = 30.00
    - Standard Deviation = 5.66
    - Minimum = 13.00
    - Maximum = 46.00
    - Interquartile Range = 8.00
    - Skewness = .011 (Standard Error = .104)
    - Kurtosis = -.137 (Standard Error = .207)
  - Women:
    - *n* = 446
    - Mean = 29.98
    - 95% Lower Bound Confidence Interval for Mean = 29.42
    - 95% Upper Bound Confidence Interval for Mean = 30.54
    - Median = 30.00
    - Standard Deviation = 6.01
    - Minimum = 13.00
    - Maximum = 45.00
    - Interquartile Range = 8.00
    - Skewness = -.068 (Standard Error = .116)
    - Kurtosis = -.251 (Standard Error = .231)
  - Total:
    - *n* = 1047
    - Mean = 29.96
    - 95% Lower Bound Confidence Interval for Mean = 29.60
    - 95% Upper Bound Confidence Interval for Mean = 30.31
    - Median = 30.00
    - Standard Deviation = 5.90
    - Minimum = 13.00
    - Maximum = 46.00
    - Interquartile Range = 8.00
    - Skewness = -.028 (Standard Error = .076)
    - Kurtosis = -.220 (Standard Error = .151)


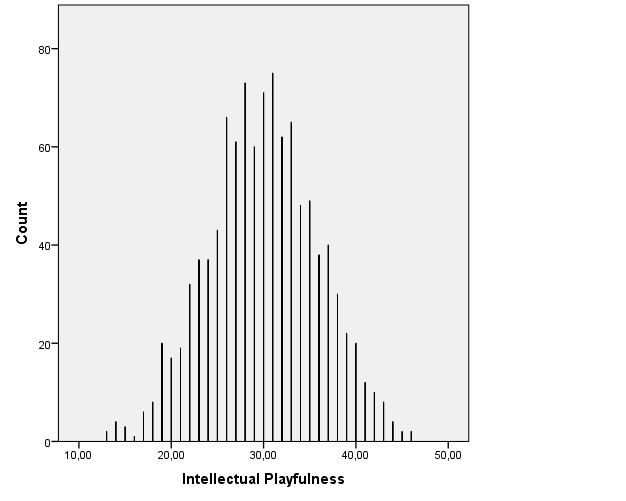


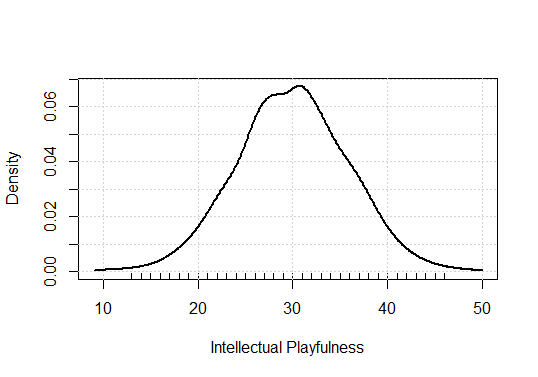


- Whimsical playfulness:


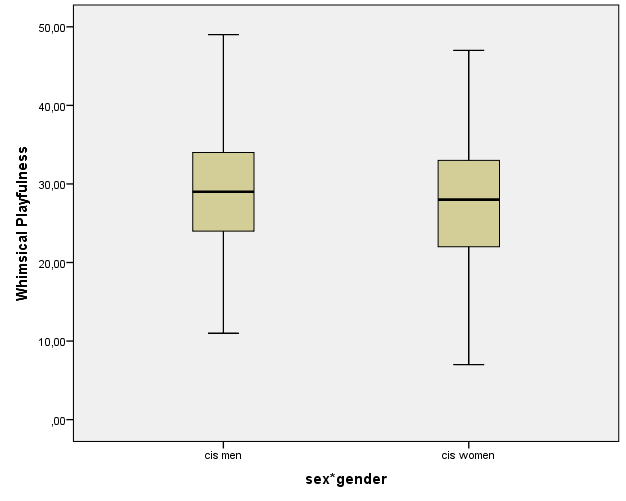


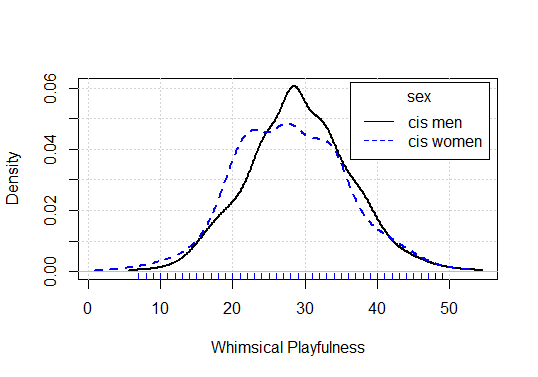


- - Men:
    - *n* = 557
    - Mean = 29.14
    - 95% Lower Bound Confidence Interval for Mean = 28.54
    - 95% Upper Bound Confidence Interval for Mean = 29.74
    - Median = 29.00
    - Standard Deviation = 7.15
    - Minimum = 11.00
    - Maximum = 49.00
    - Interquartile Range = 10.00
    - Skewness = .083 (Standard Error = .104)
    - Kurtosis = -.225 (Standard Error = .207)
  - Women:
    - *n* = 446
    - Mean = 27.89
    - 95% Lower Bound Confidence Interval for Mean = 27.17
    - 95% Upper Bound Confidence Interval for Mean = 28.61
    - Median = 28.00
    - Standard Deviation = 7.74
    - Minimum = 7.00
    - Maximum = 47.00
    - Interquartile Range = 11.00
    - Skewness = .062 (Standard Error = .116)
    - Kurtosis = -.308 (Standard Error = .231)
  - Total:
    - *n* = 1047
    - Mean = 28.69
    - 95% Lower Bound Confidence Interval for Mean = 28.24
    - 95% Upper Bound Confidence Interval for Mean = 29.14
    - Median = 29.00
    - Standard Deviation = 7.49
    - Minimum = 7.00
    - Maximum = 49.00
    - Interquartile Range = 11.00
    - Skewness = .005 (Standard Error = .076)
    - Kurtosis = -.255 (Standard Error = .151)


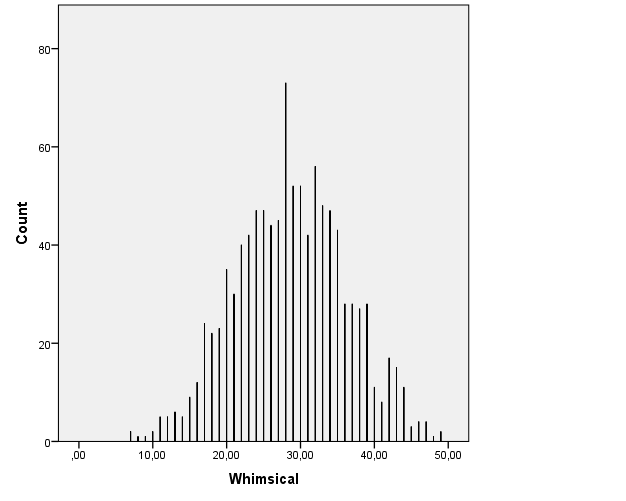


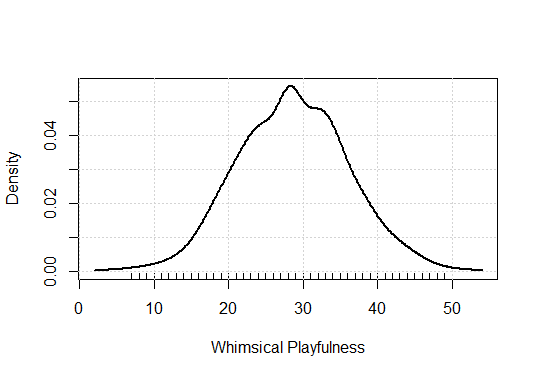


- Number of Short-term Relationships:


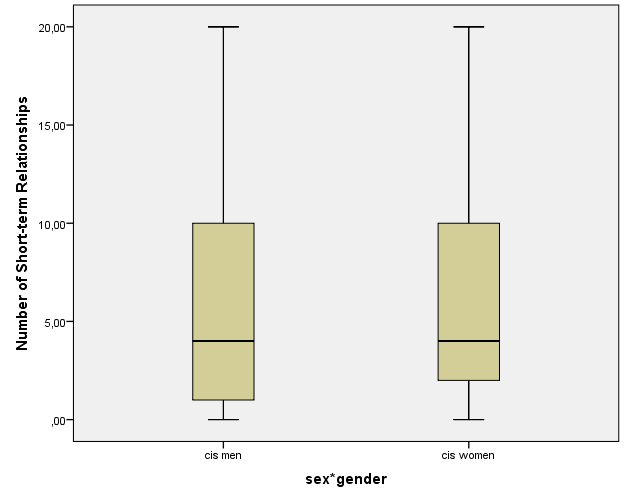


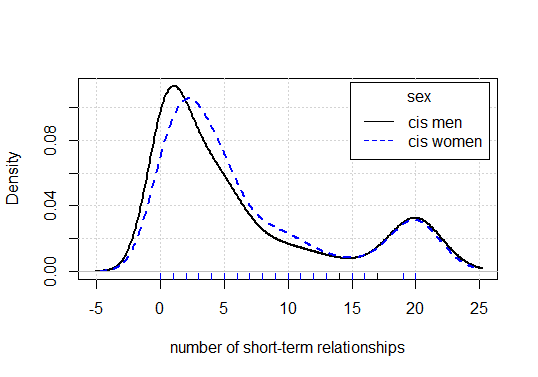


- - Men:
    - *n* = 516
    - Mean = 6.36
    - 95% Lower Bound Confidence Interval for Mean = 5.75
    - 95% Upper Bound Confidence Interval for Mean = 6.97
    - Median = 4.00
    - Standard Deviation = 7.02
    - Minimum = 0.00
    - Maximum = 20.00
    - Interquartile Range = 9.00
    - Skewness = 1.046 (Standard Error = .108)
    - Kurtosis = -.356 (Standard Error = .215)
  - Women:
    - *n* = 416
    - Mean = 6.67
    - 95% Lower Bound Confidence Interval for Mean = 6.03
    - 95% Upper Bound Confidence Interval for Mean = 7.31
    - Median = 4.00
    - Standard Deviation = 6.64
    - Minimum = 0.00
    - Maximum = 20.00
    - Interquartile Range = 8.00
    - Skewness = 1.040 (Standard Error = .120)
    - Kurtosis = -.249 (Standard Error = .239)
  - Total:
    - *n* = 969
    - Mean = 6.49
    - 95% Lower Bound Confidence Interval for Mean = 6.06
    - 95% Upper Bound Confidence Interval for Mean = 6.92
    - Median = 4.00
    - Standard Deviation = 6.88
    - Minimum = 0.00
    - Maximum = 20.00
    - Interquartile Range = 9.00
    - Skewness = 1.039 (Standard Error = .079)
    - Kurtosis = -.329 (Standard Error = .157)


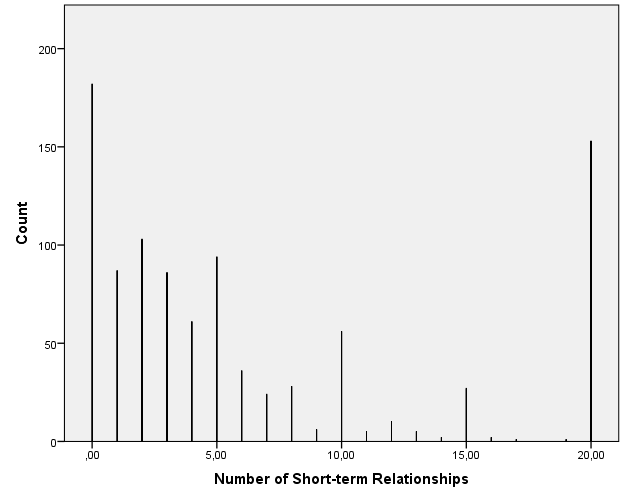


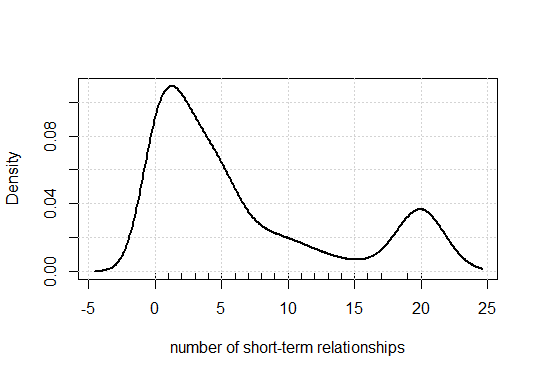


- Number of Long-term Relationships:


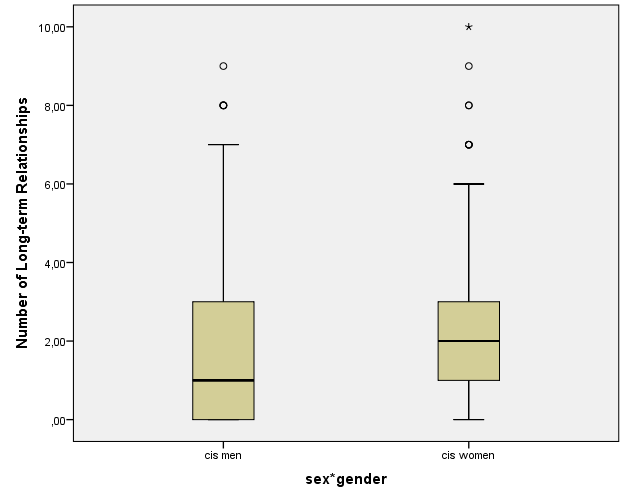


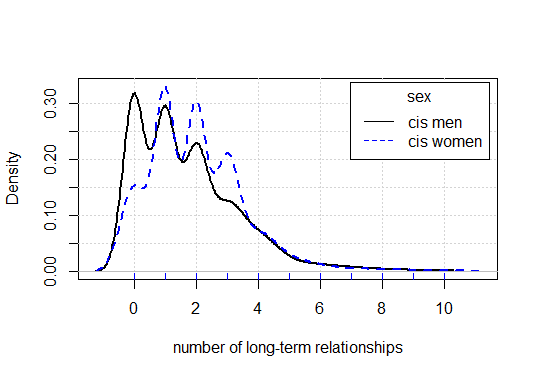


- - Men:
    - *n* = 516
    - Mean = 1.72
    - 95% Lower Bound Confidence Interval for Mean = 1.58
    - 95% Upper Bound Confidence Interval for Mean = 1.86
    - Median = 1.00
    - Standard Deviation = 1.66
    - Minimum = 0.00
    - Maximum = 9.00
    - Interquartile Range = 3.00
    - Skewness = 1.212 (Standard Error = .108)
    - Kurtosis = 1.775 (Standard Error = .215)
  - Women:
    - *n* = 416
    - Mean = 2.02
    - 95% Lower Bound Confidence Interval for Mean = 1.87
    - 95% Upper Bound Confidence Interval for Mean = 2.18
    - Median = 2.00
    - Standard Deviation = 1.59
    - Minimum = 0.00
    - Maximum = 10.00
    - Interquartile Range = 2.00
    - Skewness = 1.212 (Standard Error = .120)
    - Kurtosis = 2.723 (Standard Error = .239)
  - Total:
    - *n* = 969
    - Mean = 1.86
    - 95% Lower Bound Confidence Interval for Mean = 1.75
    - 95% Upper Bound Confidence Interval for Mean = 1.96
    - Median = 2.00
    - Standard Deviation = 1.66
    - Minimum = 0.00
    - Maximum = 10.00
    - Interquartile Range = 2.00
    - Skewness = 1.251 (Standard Error = .079)
    - Kurtosis = 2.402 (Standard Error = .157)


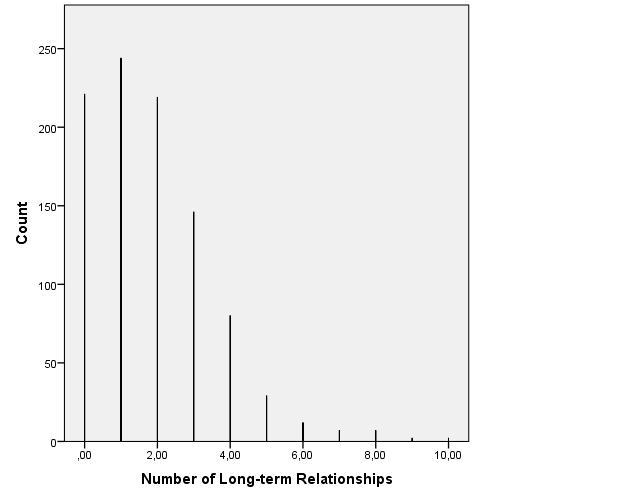


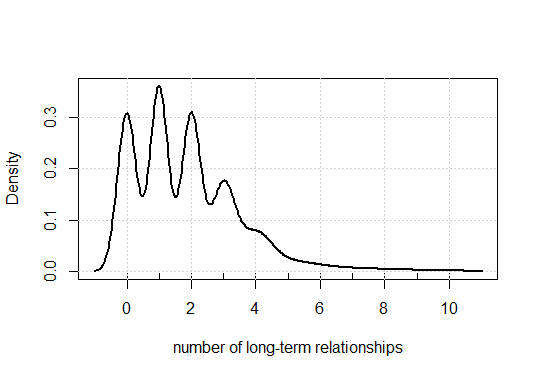

Supplement: Supplementary file 1 [file S2513843X21000190sup001.docx]
